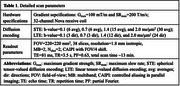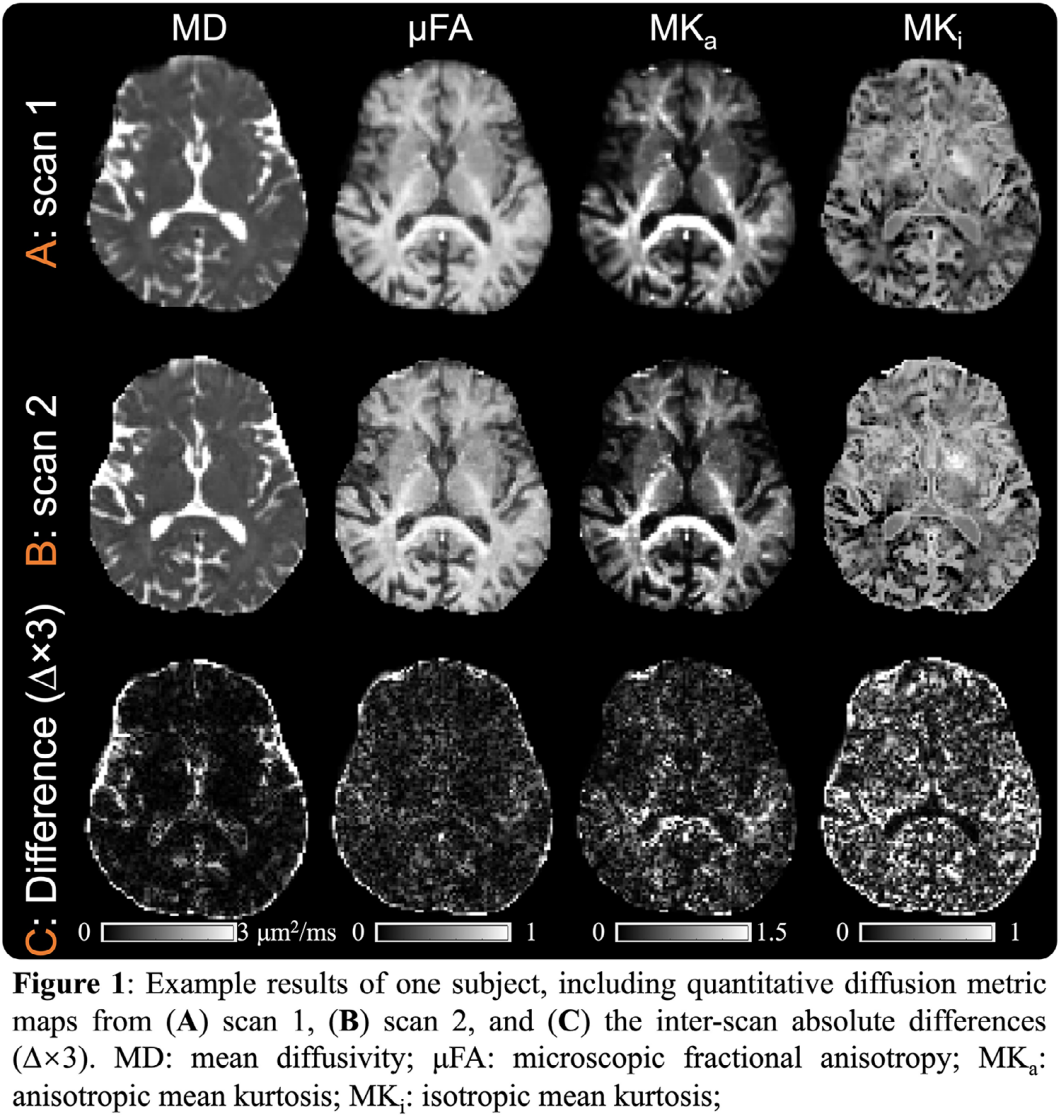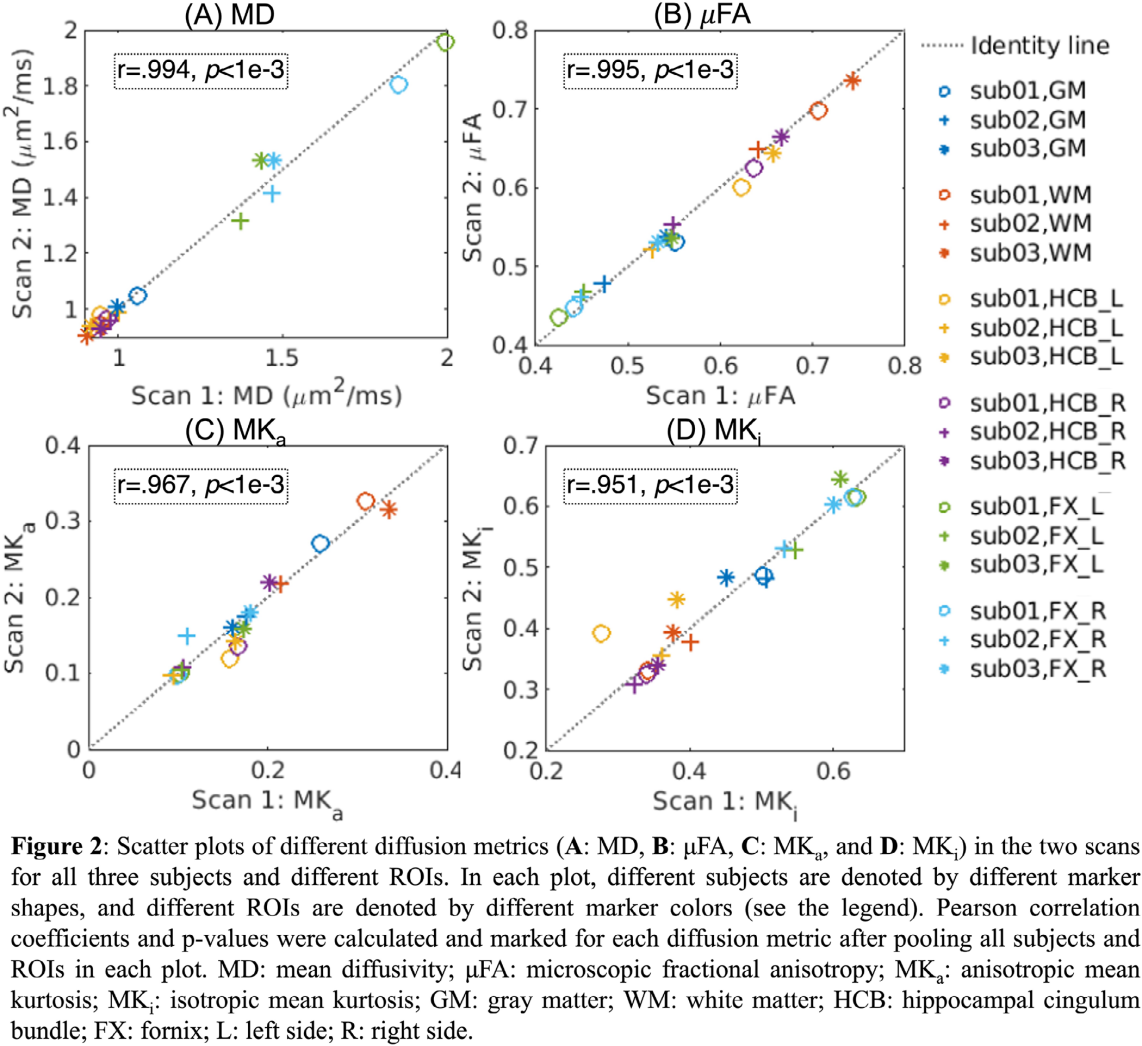# A multi‐dimensional diffusion encoding (MDE) protocol for studying brain microstructure changes in neurodegeneration

**DOI:** 10.1002/alz70862_109814

**Published:** 2025-12-23

**Authors:** Erpeng Dai, Martin K Schneider, Xuetong Zhou, S Shailja, Michael Zeineh, Jennifer A McNab

**Affiliations:** ^1^ Stanford University, Stanford, CA USA

## Abstract

**Background:**

Diffusion MRI is a promising approach to detecting brain microstructure changes associated with neurodegeneration. Unfortunately, diffusion tensor imaging (DTI) only provides limited specificity as it relies solely on single‐dimensional diffusion encoding. Multi‐dimensional diffusion encoding (MDE) is an emerging method for more specific brain microstructure mapping with advanced diffusion‐encoding waveform design and modeling. However, MDE typically decreases the diffusion‐encoding efficiency and SNR, resulting in relatively low voxel resolution (>2 mm isotropic). We aim to develop an MDE protocol of 1.8 mm isotropic with a clinically feasible scan time using an optimized MDE waveform design and an SNR‐efficient readout, for studying brain microstructure changes in neurodegeneration. Moreover, we evaluate the scan‐rescan reproducibility of the developed MDE protocol.

**Methods:**

A customized MDE sequence was developed by jointly using two different diffusion‐encoding waveforms: linear and spherical tensor‐valued diffusion encoding (LTE and STE). An SNR‐efficient multi‐band multi‐shot EPI sequence and reconstruction were used for data readout. Three cognitively normal elderly subjects (1F/2M, aged 62∼70 y/o) were scanned on a 3T GE scanner equipped with a UHP gradient coil. Detailed parameters are listed in Table 1. Quantitative diffusion metrics were calculated, including mean diffusivity (MD), microscopic fractional anisotropy (μFA), anisotropic and isotropic mean kurtosis (MK_a_ and MK_i_). Each subject was scanned twice to assess reproducibility, in the left and right hippocampal cingulum bundle and fornix (using FSL XTRACT) and global white matter/gray matter (using FSL FAST).

**Results:**

High‐quality quantitative diffusion metric maps are achieved with the developed MDE protocol (Figure 1). Pearson correlation coefficients (marked in Figure 2) range from r=0.951‐0.995 for MD, μFA, MK_a,_ and MK_i_ in the global GM and WM, left and right hippocampal cingulum and fornix (Figure 2).

**Conclusions:**

We have developed a MDE protocol of 1.8 mm isotropic and demonstrated its high scan‐rescan reproducibility, globally and in four critical WM regions for cognitive function. The achieved 1.8 mm isotropic resolution, together with a large slice coverage (∼70 mm) within a clinically feasible scan time (∼13 min), sets the foundation for more specifically mapping brain microstructure changes in critical brain regions (e.g., the medial temporal lobe) to capture neurodegeneration.